# Anti-Tumor Effects of an Oncolytic Adenovirus Expressing Hemagglutinin-Neuraminidase of Newcastle Disease Virus *in Vitro* and *in Vivo*

**DOI:** 10.3390/v6020856

**Published:** 2014-02-18

**Authors:** Dongyun He, Lili Sun, Chang Li, Ningning Hu, Yuan Sheng, Zhifei Chen, Xiao Li, Baorong Chi, Ningyi Jin

**Affiliations:** 1 Department of Gastroenterology, The First Hospital of Jilin University, Changchun 130021, China; E-Mail: spring_101@163.com; 2 Institute of Military Veterinary, Academy of Military Medical Sciences of PLA, Changchun 130122, China; E-Mails: lichang78@163.com (C.L.); 158795786@qq.com (N.H.); zjl55186666@qq.com (Y.S.); zhifeic@163.com (Z.C.); 3 Department of Gynaecology and Obstetrics, China-Japan Union Hospital of Jilin University, Changchun 130031, China; 4 Head and Neck Surgery, The Tumor hospital of Jilin province, Changchun 130001, China; E-Mail: linjiaxiaoya@163.com; 5 The Key Laboratory of Jilin Province for Zoonosis Prevention and Control, Changchun 130021, China

**Keywords:** anti-tumor, hemagglutinin-neuraminidase gene, apoptosis, oncolytic adenoviruses, esophageal cancer

## Abstract

Oncolytic virotherapy has been an attractive drug platform for targeted therapy of cancer over the past few years. Viral vectors can be used to target and lyse cancer cells, but achieving good efficacy and specificity with this treatment approach is a major challenge. Here, we assessed the ability of a novel dual-specific anti-tumor oncolytic adenovirus, expressing the hemagglutinin-neuraminidase (HN) gene from the Newcastle disease virus under the human telomerase reverse transcriptase (hTERT) promoter (Ad-hTERTp-E1a-HN), to inhibit esophageal cancer EC-109 cells in culture and to reduce tumor burden in xenografted BALB/c nude mice. *In vitro*, infection with Ad-hTERT-E1a-HN could inhibit the growth of EC-109 cells significantly and also protect normal human liver cell line L02 from growth suppression in 3-(4,5-dimethylthiazol-2-yl)-2,5-diphenyltetrazolium bromide (MTT) assays. Ad-hTERT-E1a-HN also effectively and selectively decreased the sialic acid level on EC-109 cells, but not on L02 cells. Furthermore, Ad-hTERT-E1a-HN was shown to induce the apoptosis pathway via acridine orange and ethidium bromide staining (AO/EB staining), increase reactive oxygen species (ROS), reduce mitochondrial membrane potential and release cytochrome c. *In vivo*, xenografted BALB/c nude mice were treated via intratumoral or intravenous injections of Ad-hTERT-E1a-HN. Although both treatments showed an obvious suppression in tumor volume, only Ad-hTERT-E1a-HN delivered via intratumoral injection elicited a complete response to treatment. These results reinforced previous findings and highlighted the potential therapeutic application of Ad-hTERT-E1a-HN for treatment of esophageal cancer in clinical trials.

## 1. Introduction

Esophageal cancer is one of the most common diseases in the digestive system with early metastasis, high mortality and poor prognosis. Curative treatment consists of surgical resection assisted by adjunctive therapy, such as chemotherapy and/or radiotherapy, but there is still a high rate of recurrence, leading to a five-year survival of only 25%. It has been shown that gene therapy can play a role as an adjuvant treatment modality for esophageal cancer [1]. Gene therapy is now a mature discipline, which has the potential to treat or even cure several diseases [2]. In light of the several hundred clinical trials conducted thus far, it is apparent that viral delivery methods currently represent the most appealing option for efficient gene delivery both *ex vivo* and *in vivo* [3]. Oncolytic viruses are replicating microorganisms that have been selected or engineered to grow inside tumor cells [4]. Arming oncolytic viruses with anti-cancer genes has been a major focus in cancer virotherapy, and exploited transgenes include tumor suppressor, pro-apoptotic, anti-angiogenic, “suicide,” RNA interference and immunomodulatory genes [5,6].

Newcastle disease virus (NDV) is an enveloped paramyxovirus with a single-stranded, negative-sense RNA genome. This virus has been used for the treatment of cancer patients based on its efficient replication in cancer cells, specific killing of cancer cells and its limited toxicity to normal cells [7,8]. NDV expresses two surface proteins, hemagglutinin-neuraminidase (HN) and the fusion protein. The HN protein is a 74-kDa membrane glycoprotein, which is known to boost innate immunity in anti-tumor therapy [9]. This molecule not only allows the attachment of the virus to the receptors of host cells rich in sialic acids as well as the release of viruses from the cells [10,11], but it also possesses neuraminidase activity, which can hydrolyze the sialic acid on those receptors [12]. Additionally, the HN protein plays an important role in inducing protective immunity against virus infection and is therefore susceptible to immune pressure, which generates antigenic variation [13]. Furthermore, HN can also induce IFN-ɑ and tumor necrosis factor-related apoptosis-inducing ligand (TRAIL) in human peripheral blood mononuclear cells (PBMC) and is involved in activation of apoptotic pathways [14]. All of these characteristics support HN as a promising candidate for anti-tumor therapy. 

Here, we combined the tumor-specific apoptosis-inducing gene encoding HN and a cancer-specific human telomerase reverse transcriptase promoter (hTERT) with a RAPAd.I adenovirus vector to construct a novel dual-specific anti-tumor oncolytic adenovirus Ad-hTERT-E1a-HN, as well as the control recombinant adenoviruses Ad-mock, Ad-CMV-E1a, Ad-hTERT-E1a, Ad-CMV-HN, Ad-hTERT-HN and Ad-CMV-E1a-HN. Human telomerase reverse transcriptase, a catalytic subunit of the telomerase enzyme, has been identified as an ideal tumor-associated antigen. With its broad expression in more than 85% of all cancers despite little or no expression in normal somatic cells, hTERT has been investigated as a potentially highly specific molecular target for therapeutic interventions in various types of cancers [15,16]. Therefore, hTERT has been used for tumor-specific expression of transgenes. We found that Ad-hTERT-E1a-HN could selectively target and kill tumor cells by inducing apoptosis in human esophageal cancer EC-109 cells *in vitro*. Furthermore, to determine whether Ad-hTERT-E1a-HN can cause tumor regression in BALB/c nude mice bearing EC-109 cells as xenografts, we also examined the effects of intratumoral and intravenous injections of Ad-hTERT-E1a-HN. The results indicated that Ad-hTERT-E1a-HN represents a potentially promising anti-tumor agent in developing new therapeutic strategies for the treatment of esophageal cancers and may have clinical value toward other neoplastic diseases.

## 2. Results

### 2.1. Specific Lethal Effects of Recombinant Adenoviruses in EC-109 Cells

**Figure 1:**
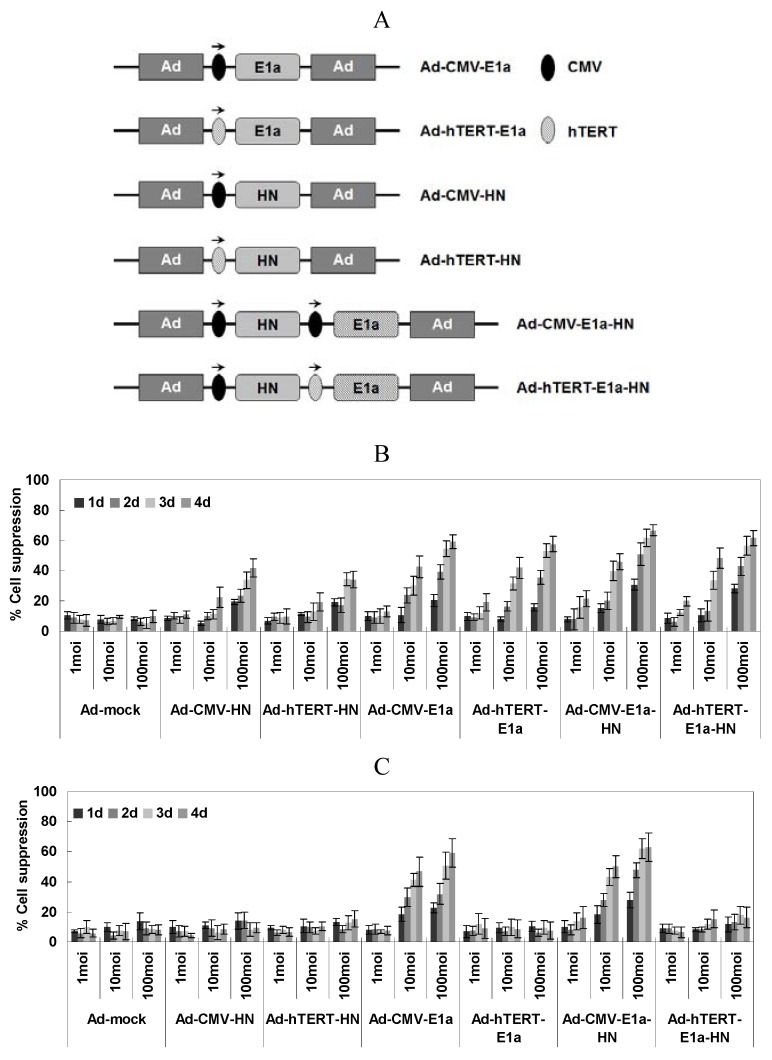
(**A**) Schematic diagram of recombinant adenoviruses. Schematic of recombinant adenoviruses depicting the organizational elements. In non-specific replication-competent adenoviruses (Ad-CMV-E1a and Ad-CMV-E1a-HN), the CMV promoter drives E1a expression. hTERT and E1a genes were incorporated in the tumor-specific replication-competent adenovirus (Ad-hTERT-E1a). HN expression from the two replication-incompetent adenoviruses lacking the E1a gene, Ad-CMV-HN and Ad-hTERT-HN, is driven by CMV and hTERT promoters, respectively, and did not replicate in either cancer cells or normal cells. In the dual-specific anti-tumor recombinant adenovirus (Ad-hTERT-E1a-HN), which demonstrated both tumor-specific replication and cell growth inhibitory effects, the hTERT promoter drives E1a and the CMV promoter drives HN expression. (E1a, essential gene for adenovirus replication; HN, specific anti-tumor gene; CMV, cytomegalovirus promoter; hTERT: tumor-specific promoter.) Selective inhibitory effects of Ad-hTERT-E1a-HN were assessed on the human esophageal cancer EC-109 cells (**B**) and normal human liver L02 cells (**C**). Effects of different MOI and infection times on viability of EC-109 cells (**B**) and L02 cells (**C**). Cells were seeded in 96-well plates (1 × 10^4^ cells/well) one day before infection with various concentrations (MOI 1, 10 and 100) of the indicated adenoviruses. Tumor viability was measured every day over a four-day period using the MTT colorimetric assay, and all measurements were performed in triplicate. Data are presented as means ± standard deviation (SD). In the EC-109 human esophageal cancer cells (**B**), Ad-CMV-E1a-HN, Ad-hTERT-E1a-HN, Ad-CMV-E1a and Ad-hTERT-E1a infection resulted in significant growth inhibition. In contrast, in L02 cells (**C**), only Ad-CMV-E1a or Ad-CMV-E1a-HN, but not Ad-CMV-HN, Ad-hTERT-HN, Ad-hTERT-E1a or Ad-hTERT-E1a-HN, inhibited cell growth.

With the aim of assessing cell viability of the recombinant adenovirus-infected EC-109 and normal human liver cell line L02, the MTT colorimetric assay was performed at various times post-infection and three different MOI of virus (Figure 1B,C). In EC-109 cells (Figure 1B), the suppression of cells by infection with replication-competent adenoviruses (Ad-CMV-E1a, Ad-hTERT-E1a, Ad-CMV-E1a-HN and Ad-hTERT-E1a-HN) increased significantly over the infection time, especially in those infected with Ad-CMV-E1a-HN or Ad-hTERT-E1a-HN. Meanwhile, there was no significant difference between cells treated with Ad-CMV-HN or Ad-hTERT-HN. Furthermore, infection with Ad-CMV-E1a-HN, Ad-hTERT-E1a-HN, Ad-CMV-E1a or Ad-hTERT-E1a at an MOI of 10 or 100 obviously inhibited cell growth compared with treatment with the replication-incompetent adenoviruses after day 4. However, there was no significant difference in the growth of recombinant adenovirus cells infected with a MOI of 1. By contrast, the growth of L02 cells infected only with non-specific replication-competent adenoviruses (Ad-CMV-E1a or Ad-CMV-E1a-HN) was significantly inhibited at a MOI of 10 or 100 compared with infection at the MOI of 1 (Figure 1C). Thus, replication-competent adenoviruses were much more effective than the replication-incompetent ones in suppressing EC-109 cells. Additionally, Ad-CMV-E1a-HN and Ad-hTERT-E1a-HN could effectively restrain the growth of cultured EC-109 cells. Compared with the inhibitory effect of Ad-CMV-E1a-HN on L02 cells, Ad-hTERT-E1a-HN could replicate specifically in EC-109 cells and selectively restrict cell growth. Interestingly, the complex relationship between infection time and MOI was synergistic, and cell viability showed a non-rigorous dependence on both factors. Therefore, we performed the following experiments after infection at MOI of 100.

### 2.2. Effects of Recombinant Adenoviruses on EC-109 Cell Membrane

**Figure 2:**
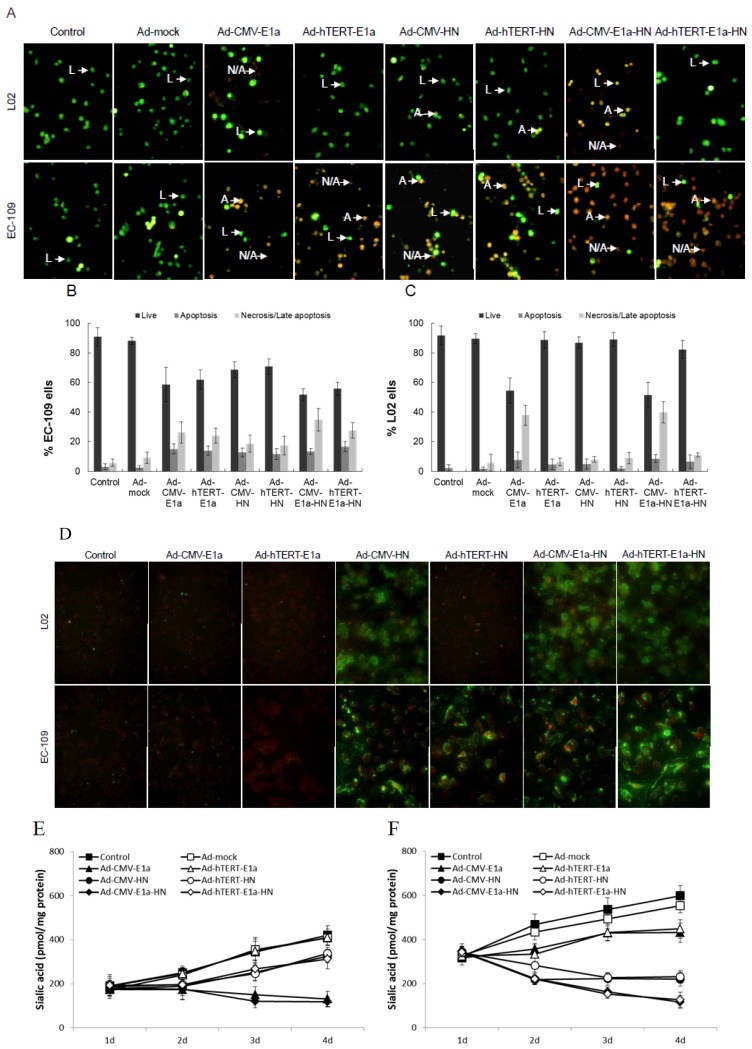
Morphological changes of EC-109 and L02 cells infected with Ad-hTERT-E1a-HN by AO/EB staining. (**A**) Fluorescence images at 100× magnification show morphological changes of recombinant adenovirus-infected EC-109 and L02 cells stained with AO/EB. IPP analysis of EC-109 (**B**) and L02 cells (**C**) infected with recombinant adenovirus was carried out to quantify proportions of live, apoptotic and necrosis/late apoptotic populations. Microscopic images were captured and analyzed by the Image-Pro Plus software program. Data are means ± standard deviation (SD). L, normal cell; A, apoptotic cell; N/A, necrosis/late apoptotic cell. (**D**) Immunofluorescence detection of HN in EC109 and L02 cells with the corresponding FITC-labeled antibody. (**E** and **F**) Sialic acid levels in EC109 and L02 cells after treatment with recombinant adenoviruses. Sialic acid levels on EC109 and L02 cells infected at a MOI 10 of recombinant adenoviruses were measured every day over a four-day period using 3,5-dihydroxytoluene, and A560 values were detected with an ultraviolet spectrophotometer. The absorbance is directly proportional to the sialic acid concentration in the sample, which was calculated with the formula: experimental A560/standard A 560 × 1.94. Data are means ± SD.

To analyze the mechanism of cell death, fluorescent assays were conducted after acridine orange (AO) and ethidium bromide (EB) staining to quantify and determine the relative percentages of live, apoptotic and necrotic EC-109 or L02 cells after infection with recombinant adenoviruses (Figure 2A–C). Chromatin condensation and nuclear fragmentation are the hallmarks of apoptotic cells. The AO dye can permeate all cells and render the nuclei and cytoplasm green, while EB stains the nucleus red, dominating over the AO stain, only when the cell membrane is disrupted [17]. As shown in Figure 2A, all recombinant adenovirus-infected EC-109 cells presented bright green and orange staining, compared with the generally green signals of healthy control and Ad-mock-treated ones. Cytotoxicity was especially strong in the Ad-CMV-E1a-HN- and Ad-hTERT-E1a-HN-treated cells. In contrast, in L02 cells, only Ad-CMV-E1a- or Ad-CMV-E1a-HN-treated cells showed bright green and orange signals. With the AO/EB method, proportions of live, apoptotic and necrosis/late apoptotic cells after recombinant adenoviruses treatment could be quantified (Figure 2B,C). The percentages of live cells in EC-109 cells infected with Ad-CMV-E1a-HN and Ad-hTERT-E1a-HN were obviously lower than those observed with recombinant adenoviruses (Figure 2B). Meanwhile, due to the loss of the tumor-specific effect of HN in L02 cells, only Ad-CMV-E1a- or Ad-CMV-E1a-HN-treated cells were cytotoxic and caused a higher proportion of apoptotic and/or necrotic cells (Figure 2C). Although all of the recombinant adenoviruses restrained the growth of EC-109 cells via induction of apoptosis and necrosis, these results indicated that Ad-hTERT-E1a-HN had the strongest anti-tumor effect.

### 2.3. Expression of HN gene and Its Effect on Sialic Acid Content in vitro

The protein expression of the HN gene was observed on infected EC-109 and L02 cells by immunofluorescence analysis with the corresponding FITC-labeled antibody. As shown in Figure 2D, the HN protein was expressed in L02 cells treated with Ad-CMV-HN, Ad-CMV-E1a-HN and Ad-hTERT-E1a-HN; meanwhile, EC-109 cells infected with Ad-CMV-HN, Ad-hTERT-HN, Ad-CMV-E1a-HN and Ad-hTERT-E1a-HN stained bright green, indicating the significant expression level of the HN gene, especially in Ad-hTERT-E1a-HN-treated cells. Furthermore, the total sialic acid content quantified using the 3,5-dihydroxytoluene method was compared between untreated cells and those of EC-109 or L02 cells treated with the various recombinant adenoviruses. In L02 cells (Figure 2E), the sialic acid content of Ad-CMV-E1a- and Ad-CMV-E1a-HN-treated cells gradually decreased over the infection time, while cells infected with other recombinant adenoviruses retained significantly higher levels of sialic acid on the fourth day compared with those on the first day. In contrast, in EC-109 cells (Figure 2F), at 24 h after infection with recombinant adenoviruses, no significant difference in the content of total sialic acid was found among the groups. Interestingly, at day 2 after infection, the total sialic acid levels of cells treated with Ad-CMV-HN, Ad-hTERT-HN, Ad-CMV-E1a-HN and Ad-hTERT-E1a-HN began to decrease, while others groups showed a rising trend of the total sialic acid content. At a later time point (ay 3 post-infection), Ad-mock-infected and control groups contained significantly higher levels of sialic acid compared with the Ad-CMV-E1a and Ad-hTERT-E1a groups, but Ad-CMV-HN-, Ad-hTERT-HN-, Ad-CMV-E1a-HN- and Ad-hTERT-E1a-HN-treated cells had a notable decrease, especially the Ad-CMV-E1a-HN and Ad-hTERT-E1a-HN groups. By day 4 post-infection, the sialic acid levels of the Ad-CMV-E1a-HN and Ad-hTERT-E1a-HN treatment groups were almost eliminated. While the sialic acid levels in the Ad-CMV-HN and Ad-hTERT-HN groups decreased, they did not change significantly between the third and fourth day post-infection. However, the concentrations of sialic acid of the control group and the Ad-mock-, Ad-CMV-E1a- and Ad-hTERT-E1a-treated cells gradually increased, which was accompanied by cell proliferation. These results suggested that Ad-hTERT-E1a-HN could effectively decrease the sialic acid level in EC-109 cells and would be suitable as an anti-tumor treatment.

### 2.4. Effects of Recombinant Adenoviruses on Signaling Molecules in EC-109 Cells

**Figure 3:**
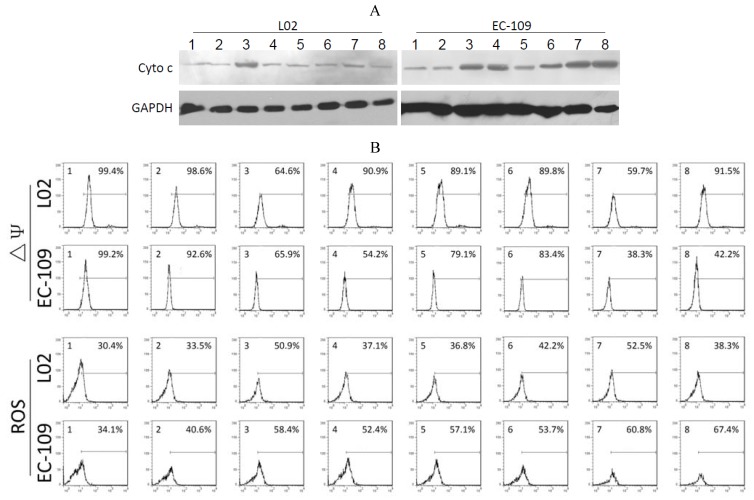
Analysis of mitochondrial permeability transition of the recombinant adenovirus-treated EC-109 cells and L02 cells. (A) Expression of cytochrome c in recombinant adenovirus-treated EC-109 and L02 cells was detected by Western blot. (B) Flow cytometric determination of ΔΨm and ROS. The proportions were calculated as follows: number of stained cells/total number of cells (%). 1. Control; 2. Ad-mock; 3. Ad-CMV-E1a; 4. Ad-hTERT-E1a; 5. Ad-CMV-HN; 6. Ad-hTERT-HN; 7. Ad-CMV-E1a-HN; 8. Ad-hTERT-E1a-HN.

As shown in Figure 3A, significant quantities of cytochrome c were detected in the cytosol of Ad-CMV-E1a-, Ad-hTERT-E1a-, Ad-CMV-E1a-HN- and Ad-hTERT-E1a-HN-infected EC-109 cells. The levels of cytochrome c in cells treated with Ad-CMV-E1a-HN and Ad-hTERT-E1a-HN were higher than in Ad-CMV-E1a- and Ad-hTERT-E1a-treated groups. However, only Ad-CMV-E1a- and Ad-CMV-E1a-HN-treated L02 cells showed a small amount of released cytochrome c, while other recombinant adenovirus had no significant effects in the L02 cells. As shown in Figure 3B, significant ΔΨm losses were detected in Ad-CMV-E1a-HN- (38.3%) and Ad-hTERT-E1a-HN-infected (42.2%) EC-109 cells, but not in the cells treated with the other recombinant adenoviruses. The slight decrease in ΔΨm was also detected in Ad-CMV-E1a- (65.9%) and Ad-hTERT-E1a-infected (54.2%) cells. In L02 cells, only the Ad-CMV-E1a and Ad-CMV-E1a-HN treatment resulted in a ΔΨm loss of 64.6% and 59.7%, respectively, and the ΔΨm values of L02 cells treated with other recombinant adenovirus were similar to that of the untreated group. 

The various recombinant adenoviruses showed differential abilities to elevate levels of ROS (Figure 3B). EC-109 cells infected with Ad-CMV-E1a-HN and Ad-hTERT-E1a-HN showed a significant increase in ROS of 60.8% and 67.4%, respectively, while in recombinant adenovirus-treated L02 cells, only Ad-CMV-E1a and Ad-CMV-E1a-HN could raise the levels of ROS by 50.9% and 52.5%, respectively. These experiments indicated that the apoptotic pathway in EC-109 cells triggered by Ad-hTERT-E1a-HN was associated with the release of cytochrome c, loss of ΔΨm and increase of ROS. Compared with other recombinant adenovirus, Ad-hTERT-E1a-HN showed a distinct level of tumor-specific targeting and killing. 

### 2.5. Effects of Recombinant Adenoviruses on EC-109 Cell-Derived Tumors *in Vivo*

**Figure 4:**
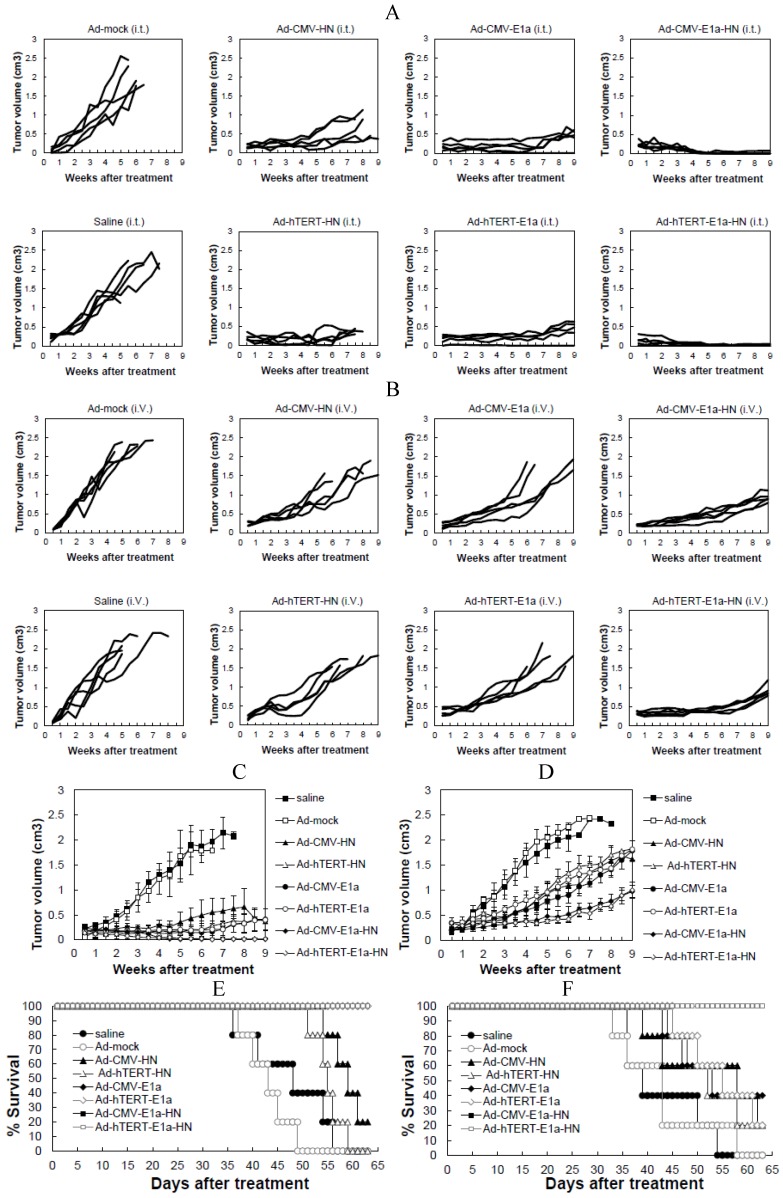
Effects of recombinant adenovirus on tumors established by xenografting EC-109 cells in BALB/c nude mice. (**A**) Tumor growth kinetics of mice that received intratumoral injections of adenovirus; (**B**) Tumor growth kinetics of mice that received intravenous injections adenovirus; (**C**) Mean tumor volumes in intratumorally injected groups; (**D**) Mean tumor volumes in intravenous injections groups; (**E**) Survival curve of mice treated intratumorally; (**F**) Survival curve of mice treated intravenously. The day of the first injection was considered day 0. Data are presented as means ± SD.

To evaluate the anti-tumor potential of the vectors *in vivo*, we treated tumor-bearing mice with recombinant adenoviruses via intratumoral or intravenous injections. Each tumor was measured twice a week, and all animals in the study were sacrificed after nine weeks. The growth kinetics of the tumors treated with intratumoral injections are shown in Figure 4A. Compared with the saline control, Ad-mock- and Ad-CMV-HN-infected groups,other groups infected with recombinant adenoviruses showed suppression of tumor growth. Treatment of mice with Ad-CMV-E1a-HN and Ad-hTERT-E1a-HN induced a potent anti-tumor response, especially in the Ad-hTERT-E1a-HN-infected group, in which the tumor volume was significantly reduced and close to regression. By contrast, the Ad-hTERT-HN-, Ad-CMV-E1a- and Ad-hTERT-E1a-infected tumors slowly grew and gradually resumed their growth at six weeks. In mice injected via the intravenous route (Figure 4B), tumors of the Ad-CMV-E1a-HN- and Ad-hTERT-E1a-HN-infected groups decreased most significantly in size, compared with the saline control, Ad-mock- and Ad-mock-infected groups. Growth of the Ad-CMV-E1a-, Ad-hTERT-E1a-, Ad-CMV-HN- and Ad-hTERT-HN-treated tumors were also suppressed, compared with saline-treated and Ad-mock-infected groups; however, there was no significant difference in growth rates of the tumors in these groups. In the intratumorally injected groups, the mean tumor volumes of the recombinant adenovirus treatment groups were reduced obviously and were basically always maintained at a lower level, compared with saline-treated and Ad-mock-infected groups (Figure 4C). Notably, the tumors nearly disappeared in the Ad-hTERT-E1a-HN-infected group. The results from Figure 4A,C indicated that the intratumoral injection of Ad-CMV-E1a-HN or Ad-hTERT-E1a-HN could reduce tumor volume efficiently. 

Mean tumor volumes in the intravenously injected groups were also evaluated (Figure 4D). Although the tumors infected with Ad-CMV-E1a-HN and Ad-hTERT-E1a-HN gradually resumed their growth after two weeks, their tumor volumes were the lowest compared with other groups. The tumor volumes of the Ad-CMV-E1a-, Ad-hTERT-E1a-, Ad-CMV-HN- and Ad-hTERT-HN-infected groups were reduced compared with those of the saline-treated and Ad-mock-infected groups, while there were no significant differences between the groups. As shown in Figure 4B,D, the efficacy in intratumorally injected groups was better than that of the intravenously injected groups. The main reason for this difference may be that the adenoviruses delivered by direct intratumoral injection could produce a more rapid effect than those by intravenous injection. Ad-CMV-E1a-HN- and Ad-hTERT-E1a-HN-treated mice could reduce tumor burdens more significantly than other recombinant adenoviruses. 

We also evaluated the lifespan of mice following treatment. As shown in Figure 4E, when the injections were performed intratumorally, infection with Ad-CMV-E1a, Ad-hTERT-E1a, Ad-CMV-E1a-HN or Ad-hTERT-E1a-HN significantly improved the mean survival time, while the saline-treated and Ad-mock-, Ad-CMV-HN- and Ad-hTERT-HN-infected groups had lower mean survival times of 48.5 days, 43.5 days, 59.5 days and 55.5 days, respectively. These results demonstrate that intratumoral injection of replication-competent adenoviruses could improve survival effectively in mice. 

In the intravenously injected groups, Ad-CMV-E1a-HN and Ad-hTERT-E1a-HN could increase survival rates of nude mice to the mean survival time of 63 days, which was significantly better than that observed in Ad-CMV-HN-, Ad-hTERT-HN-, Ad-CMV-E1a- and Ad-hTERT-E1a-infected groups (Figure 4F). As expected, saline-treated and Ad-mock-infected mice had the worst survival rates with mean survival times of only 39.5 days and 43.25 days, respectively. The results indicate that intravenous injections of Ad-CMV-E1a-HN and Ad-hTERT-E1a-HN conferred significant survival benefits *in vivo*.

### 2.6. Discussion

Cancer is a major cause of death worldwide. Although significantly improved over recent decades, the efficacy of tumor treatments is still limited. Thus, developing novel therapeutic strategies for cancer patients remains a constant need. Oncolytic adenoviruses are promising tools in cancer therapeutics due to their ability to be genetically manipulated and exhibit multiple distinct anti-cancer mechanisms, including direct lysis, apoptosis induction, expression of toxic proteins, autophagy and shutting-down of protein synthesis, as well as the induction of anti-tumoral immunity [5]. Assessing various therapeutic genes to insert into the viral genome has been a major focus in cancer virotherapy, and types of transgenes thus far considered for this purpose include tumor suppressor, pro-apoptotic, anti-angiogenic, “suicide” and immunomodulatory genes [5]. Xiao-Ping He *et al.* [18] previously showed that the anti-tumor effect of a conditionally replicating adenovirus (CRAd) vector modified by incorporation of an anti-angiogenesis inhibitor gene (CRAd-Cans) was even more potent than that of the replication-deficient adenovirus Ad5-Cans against pancreatic cancer both *in vivo* and *in vitro*. Ji *et al.* [19] suggested that hTERT promoter-driven oncolytic CRAd vector in combination with HSV tk /GCV gene therapy could effectively reduce growth of human retinoblastoma in an orthotopic nude mouse model but not in primary human retinal pigment epithelial cells (hRPE). Lin Fang *et al.* [20] inserted a novel 720-bp truncated minimal E1a gene (mE1a) and hTERT into an oncolytic adenoviral vector lacking the E1b gene. The constructed vector was shown to infect and replicate selectively with high efficiency and exerted an effective anti-tumor activity in human cancer cell lines as well as in hepatocarcinoma (HepG II) xenografted nude BALB/c mice [20]. 

In the present study, we constructed a novel dual specific anti-tumor oncolytic adenovirus Ad-hTERT-E1a-HN by inserting NDV HN gene and hTERT promoter into a RAPAd.I adenovirus vector, as well as the control recombinant adenoviruses (Figure 1A). Furthermore, we evaluated the anti-tumor effects of these novel oncolytic viruses on esophageal cancer *in vitro* and *in vivo*. In order to demonstrate that Ad-hTERT-E1a-HN selectively replicated in human EC-109 tumor cells, but not in normal cells, we also used Ad-hTERT-E1a-HN to infect the human L02 cells. With the extension of infection times and the increase of the infective dose (a MOI of 1, 10 or 100), the inhibitory effects on EC-109 cells treated with Ad-CMV-E1a-HN and Ad-hTERTE1a-HN became more obvious than those with other recombinant adenoviruses. In L02 cells, the inhibition was still obvious in Ad-CMV-E1a-HN-treated cells, compared with the slight inhibitory effect in the Ad-hTERT-E1a-HN-treated group. The results showed that Ad-hTERT-E1a-HN could replicate and restrict growth specifically in EC-109 cells (Figure 1B,C). Furthermore, cell viability showed a non-rigorous dependence on both infection time and MOI.

Apoptosis is a cell death program that eliminates harmful and severely damaged cells and maintains tissue homeostasis in multicellular organisms [21]. A number of viruses have the ability to induce apoptosis and contribute to cytopathic effects in infected cells [22]. NDV is a tumor selective and intrinsically oncolytic avian paramyxovirus that has been considered as a potentially powerful tool for cancer therapy [23]. Several studies have shown that NDV can induce apoptosis in various cancer cell types by activating the mitochondrial death pathway (intrinsic pathway) and the death receptor pathway (extrinsic pathway) [24,25,26], ultimately inducing caspase-dependent pathways in infected cells that lead to the biochemical and morphological changes characteristic of apoptosis [22,27]. To avoid the side effect after treatment with the virus alone, we were interested in studying the anti-tumor mechanism of apoptosis induction by HN. In the present study, we primarily investigated the effects of Ad-hTERT-E1a-HN on cell surface sialic acid levels, which are associated with tumor cell behavior, such as invasiveness and metastasis. The results showed that Ad-hTERT-E1a-HN could effectively decrease the sialic acid level on EC-109 cells, while it did not affect the total sialic acid content in L02 cells. The AO/EB staining assay used in this study to analyze cell death pathways could quantify the relative proportions of live, apoptotic and necrotic recombinant adenoviruses-infected cells (Figure 2A–C). The results indicated that Ad-hTERT-E1a-HN could significantly restrain the growth of EC-109 cells via induction of apoptosis and necrosis, while it did not affect the normal L02 cells. Furthermore, Ad-hTERT-E1a-HN was found to cause the apparent increase of reactive oxygen species (ROS), and also significantly reduce the ΔΨm and release of cytochrome c in EC-109 cells. By contrast, these effects of Ad-hTERT-E1a-HN on L02 cells were minor. Therefore, compared with Ad-CMV-E1a-HN and other recombinant adenoviruses, Ad-hTERT-E1a-HN had the potential ability to specifically kill tumor cells by inducing the apoptosis pathway.

Anti-tumor activities of the recombinant adenoviruses were also evaluated in a human esophageal cancer xenograft mouse model, which further confirmed the efficacies observed *in vitro*. Esophageal cancer is considered one of the most refractory pernicious malignant tumors with a propensity for local progression and distant dissemination. Despite ongoing research in the treatment of esophageal cancers, the prognosis for long-term survival remains poor. A five-year overall survival for patients undergoing resection for esophageal cancer was reported to be between 25% and 39%, depending on surgical modality and the use of neoadjuvant therapy [28]. In order to improve the poor prognosis, many strategies have been implemented. Ye *et al.* [29] used curcumin, (-)-epigallocatechin-3-gallate (EGCG), lovastatin and their combinations to treat esophageal cancer TE-8 and SKGT-4 cells. Although all of these treatments were found to significantly reduce the viability and invasion capacity of esophageal cancer cells *in vitro*, they were much less effective when tested *in vivo* in nude mouse xenografts, especially curcumin or lovastatin used individually [30]. Papineni *et al.* [31] also examined the effects of daily administration of tolfenamic acid (TA, 20 mg/kg/day) on tumor growth in athymic nude mice bearing SEC-1 cells as xenografts. Although the results showed that this dose of TA could significantly inhibit tumor growth and tumor weight, at the same time it increased apoptosis and decreased Sp1 and c-Met staining in tumors from treated mice; however, TA did not achieve a complete response *in vivo* using an animal model of esophageal cancer [31]. In our study, effects of various recombinant adenoviruses were evaluated via intratumoral (A) and intravenous (B) injections into BALB/c nude mice xenografted with esophageal cancer EC-109 cells. Ad-hTERT-E1a-HN was found to have significant anti-tumor effects compared with those of other recombinant adenoviruses. Injection of Ad-hTERT-E1a-HN directly into tumors resulted in a complete response to treatment (Figure 4A,C) and the longest mean survival time of 63 days, which were the most optimal outcomes compared with results from other recombinant adenovirus-treated groups (Figure 4E). When infection was carried out intravenously, Ad-hTERT-E1a-HN did not lead to complete tumor regression, although it could still suppress the growth of the tumor (Figure 4B,D) and significantly increase the survival rate of nude mice (Figure 4F), compared with other groups. We demonstrated that the efficacy of intratumoral injection was greater than that of intravenous injection, primarily due to the direct deposition of the adenovirus at the tumor site which facilitated infection and rapid induction of anti-tumor effects. Furthermore, we did not observe any toxic effects after injection of Ad-hTERT-E1a-HN during the *in vivo* experiments described here. These encouraging results showing the curative effectiveness of Ad-hTERT-E1a-HN indicated that it will be a potentially highly potent, effective and safe anticancer agent. 

## 3. Experimental Section

### 3.1. Cell Lines and Animals

The human esophageal cancer cell line EC-109 and normal human liver cell line L02 were obtained from the China Center for Type Culture Collection, Chinese Academy of Sciences (Shanghai, China). The cells were maintained in Roswell Park Memorial Institute (RPMI) 1640 and Dulbecco’s modified Eagle media (DMEM) complete medium (Gibco, Carlsbad, CA, USA) which contained 10% fetal bovine serum (FBS: Gibco), 100 U/mL penicillin and 100 μg/mL streptomycin at 37 °C in 5% CO_2_, respectively. All cell lines were passaged for no more than 6 months after receipt. The cells were routinely subcultured every 2–3 days and were all taken from the logarithmic phase of growth. Female BALB/c nude mice at 6–8 weeks old were purchased from the Experimental Animal Center of the Academy of Military Medical Sciences (Beijing, China) and bred under Chinese government guidelines.

### 3.2. Recombinant Adenoviruses

The dual-specific anti-tumor oncolytic adenovirus Ad-hTERT-E1a-HN, as well as the control recombinant adenoviruses Ad-mock, Ad-CMV-E1a, Ad-hTERT-E1a, Ad-CMV-HN, Ad-hTERT-HN, Ad-CMV-E1a-HN were constructed and characterized (Figure 1A) previously [32,33]. Briefly, shuttle plasmids of recombinant adenoviruses were co-transfected into HEK-293 cells where the recombinant adenoviruses were obtained by homologous recombination. Purification of the virus was performed using the Adeno-X Virus Purification kit (BD Bioscience Clontech, Mountain View, CA, USA). Titers of recombinant virus were determined by plaque assays and expressed as plaque-forming units (pfu) per milliliter of virus suspension [34].

### 3.3. Indirect Immunofluorescence Assay

Indirect immunofluorescence assays were performed to identify the expression of the HN gene as described previously [33]. EC-109 and L02 cells incubated on slides were infected with the recombinant adenovirus at a MOI of 100. After cytopathic effects were observed, the slides were fixed in acetone at 4 °C for 5 min and then blocked with 5% skimmed milk in Tris-buffered saline for 2 h. Slides were incubated with an NDV antibody (with 0.05% Evans Blue) for 2 h followed by incubation with goat anti-chicken IgY antibody labeled with fluorescein isothiocyanate (FITC) (with 0.05% Evans Blue) for 2 h. After washing with phosphate-buffered saline (PBS) two times, images of representative cells were captured with a charged-coupled device (CCD) connected to a fluorescence microscope VANOX (Olympus, Tokyo, Japan). Ad-mock-infected cells were used as the negative control.

### 3.4. MTT Colorimetric Assay

The MTT (3-[4,5-dimethylthiazol-2-yl]-2,5-diphenyltetrazolium bromide; thiazolyl blue, Sigma, St. Louis, MO, USA) colorimetric assay was performed to detect cell viability [34]. In brief, EC-109 and L02 cells were seeded in 96-well plates (1 × 10^4^ cells/well) at 37 °C in 5% CO_2_ for 24 h. The cells were then infected with various concentrations (a MOI of 1, 10 and 100) of recombinant adenovirus. Cell viability was measured every day over a four-day period by treating cells with 20 µL MTT (5 mg/mL) and incubating for 4 h at 37 °C to allow MTT metabolization. The culture medium was removed, and the crystals formed were dissolved in 150 µL of dimethylsulfoxide. The absorbance was measured at 490 nm with an enzyme-linked immunosorbent assay (ELISA) Sunrise plate reader (Tecan, Mannedorf, Switzerland). The percentage of cell death induced by the recombinant adenovirus was expressed using the following formula: [100 × (absorbance value of control cells-absorbance value of experimental cells)/(absorbance value of control cells)]. Untreated EC-109 and L02 cells were used as controls, and all measurements were performed in triplicate. 

### 3.5. Acridine Orange (AO)/Ethidium Bromide (EB) Staining Assay

The AO and EB staining assay was performed as described previously to determine the relative percentages of live, necrotic and apoptotic cells [34]. EC-109 and L02 cells that had been infected with the recombinant adenoviruses for 48 h were trypsinized and washed three times in Hank’s balanced salt solution (HBSS). An aliquot of 250 μL of EC-109 cells or L02 cells was placed in a microcentrifuge tube, and 2 µL of EB and 2 µL of AO were added. After vortexing, 20 μL of the sample was placed on a microscope slide with cover slip, and images of the representative cells were captured using a fluorescence microscope (Olympus, Tokyo, Japan). Images from the microscope were captured with the Image-Pro Plus (IPP, version 5.0.2) [35] software program and analyzed the following day. Tests were performed in triplicate, and non-infected cells were used as control. At least 500 cells were measured from each sample to determine the presence of normal, apoptotic or necrotic chromatin. 

### 3.6. Sialic Acid Content Assay

The 3,5-dihydroxytoluene (Sigma) method was used for the quantitative determination of total sialic acid abundance as described previously [33]. Sialic acid levels in the recombinant adenovirus-infected EC-109 and L02 cells (1 × 10^6^ cells/well) were measured every day over a four-day period. In brief, after 48 hr of infection with a MOI of 100 of recombinant adenoviruses, EC-109 and L02 cells were collected and resuspended in 500 µL double distilled water. Sialic acid content was then determined by using 3,5-dihydroxytoluene, and A560 values were detected with an ultraviolet spectrophotometer. The absorbance is directly proportional to the sialic acid concentration in the sample, which is determined with the following formula: experimental A560/standard A 560 × 1.94 [33]. Non-infected EC-109 and L02 cells were used as controls. 

### 3.7. Western Blot Analysis

EC-109 and L02 cells were infected with recombinant adenoviruses for 48 h. The extracts of cytochrome c were analyzed by Western blot as described previously [34]. Samples were separated by 12% sodium dodecyl sulfate-polyacrylamide gel electrophoresis (SDS-PAGE) and electroblotted onto Hybond-C membranes (Amersham, Piscataway, NJ, USA). The membranes were immersed in 5% milk in Tris-buffered saline (TBS). Blots were incubated with a cytochrome c rabbit monoclonal antibody (Cell Signaling, Danvers, MA, USA) for 2 h, and the secondary antibody was an anti-mouse antibody labeled with horseradish peroxidase (Cell Signaling) for 2 h. Signals were visualized via the enhanced chemiluminescence (ECL) Western blotting substrate kit (Pierce, Rockford, IL, USA). Extracts of uninfected cells were used as the negative control, and detection of glyceraldehyde 3-phosphate dehydrogenase (GAPDH) was used as the internal control.

### 3.8. Measurement of Mitochondrial Membrane Potential (ΔΨm)

The laser dye rhodamine 123 (Rho123, Sigma) was used to detect mitochondrial permeability transition. Briefly, the recombinant adenovirus-infected EC-109 and L02 cells (1 × 10^6^ cells/well) were trypsinized and transferred to a microfuge tube. After centrifugation at 3,000 rpm, 4 °C for 5 min, 10 µL of Rho123 was added to each sample, which was then incubated at 37 °C for 30 min. Thereafter, samples were washed with PBS three times. Fluorescence signals were detected by flow cytometry (FACScan, BD Biosciences, San Jose, CA, USA). The ΔΨm was quantified by determining the decrease in fluorescence of the cells. Untreated EC-109 and L02 cells were used as controls.

### 3.9. Reactive Oxygen Species (ROS) Assay

Various pathologies can result from oxidative stress-induced apoptotic signaling due to increased ROS [36]. To quantify intracellular ROS, 2',7'-dichlorfluorescein-diacetate (DCFH-DA) was used in the study. Briefly, EC-109 and L02 cells (1 × 10^6^ cells/well) were infected with recombinant adenoviruses for 48 hr. Uninfected cells were used as the negative control. ROS was determined by treating the cells with 10 µmol/L DCFH-DA at 37 °C for 30 min and then washing with PBS three times. Data were analyzed by flow cytometry (FACScan, BD, Franklin Lakes, AJ, USA).

### 3.10. Animal Experiments

EC-109 cells were trypsinized and resuspended in serum-free Hank’s balanced salt solution (HBSS). The number of viable cells was determined by trypan blue exclusion, and the cell concentration was adjusted to 1 × 10^6^ cells/mL. One hundred microliters of the cell suspension (1 × 10^5^) per flank was implanted subcutaneously into the right flanks of the BALB/c nude mice. After establishment of the models with tumors reaching a diameter of 3–5 mm, the tumor-bearing mice were randomly divided into two independent experimental sets, including the intratumoral injection set (A) and the intravenous injection set (B). Each set contained eight groups (five mice per group), which were given either (1) saline; (2) Ad-mock; (3) Ad-CMV-HN; (4) Ad-hTERT-HN; (5) Ad-CMV-E1a; (6) Ad-hTERT-E1a; (7) Ad-CMV-E1a-HN; or (8) Ad-hTERT-E1a-HN. Mice in set (A) received intratumoral injections of various recombinant adenoviruses at a dose of 1 × 10^8^ pfu in saline per mouse, and the control group received saline alone. In set (B), the injections were performed via the tail vein with various recombinant adenoviruses at the same dose as above. The injections were given every 10 days for a total of 4 times. Tumor size was measured using calipers twice a week, and tumor volumes were calculated as follows: [0.52 × (smallest diameter of tumor)^2^ × (largest diameter of tumor)] [34]. All animals were monitored daily and sacrificed 9 weeks after the first immunization.

### 3.11. Statistical Analysis

Statistical significance of differences was calculated using one-way analysis of variance (ANOVA), and *p* values less than 0.05 were considered statistically significant. The survival rates were calculated by Log rank tests. Data from all animals are represented in Kaplan-Meier plots.

## 4. Conclusions

In summary, we explored the potential effects of Ad-hTERT-E1a-HN on esophageal cancer EC-109 cells *in vitro* and *in vivo*. The results demonstrated that Ad-hTERT-E1a-HN specifically replicated in human EC-109 tumor cells and restricted the growth of these cells selectively while showing no adverse effects on L02 cells. Furthermore, we found that Ad-hTERT-E1a-HN could potentially induce the apoptosis pathway, causing the apparent increase of reactive oxygen species (ROS), reducing the ΔΨm and releasing significant amounts of cytochrome c from the mitochondria. In addition, the *in vivo* anti-tumor experiment showed that Ad-hTERT-E1a-HN could significantly suppress tumor growth, especially via intratumoral injection, which elicited a complete response to treatment and conferred the longest mean survival time of 63 days observed in this study. All these features highlight the need for further evaluation of Ad-hTERT-E1a-HN in a novel approach for clinical treatment of esophageal cancer.
